# Synchronous multiple primary early stage esophageal cancers diagnosed in a teenager using confocal laser endomicroscopy

**DOI:** 10.1055/a-2599-6761

**Published:** 2025-05-14

**Authors:** Jing Liu, Mu Cai, Mei Yang, Jun Luo, Qiu Zhao

**Affiliations:** 1 89674 Department of Gastroenterology, Zhongnan Hospital of Wuhan University, Wuhan, China; 2 650074 Department of Gastroenterology, Ezhou Central Hospital, Ezhou, Hubei, China; 3 89674 Department of Pathology, Zhongnan Hospital of Wuhan University, Wuhan, China

Esophageal cancer predominantly affects the elderly and is often overlooked in the young, resulting in patients presenting at advanced disease stages with poorer survival 1 . Early diagnosis is pivotal. Confocal laser endomicroscopy (CLE) holds significant promise in diagnosing and monitoring early upper gastrointestinal tract cancers. In a previous study, we reported the use of CLE to image a gastric adenocarcinoma, fundic gland type 2 , thereby aiding diagnosis.

We now report the case of a 16-year-old boy with a 1-year history of intermittent epigastric pain who underwent an esophagogastroduodenoscopy (EGD) that revealed multiple esophageal lesions. The EGD identified two 0-IIb lesions at 23–25 cm and 30 cm from the incisors, featuring patchy, reddish mucosa with clear borders ( Fig. 1 **a, b** ). Magnification endoscopy with narrow-band imaging (ME-NBI) showed brownish, regularly patterned lesions, classified as B1 according to the Japanese Society of Esophagus criteria ( Fig. 1 **c, d** ). Lugol iodine chromoendoscopy revealed Lugol-voiding lesions, with a pink sign later transitioning to a bright silver sign under NBI ( Fig. 1 **e–h** ). CLE demonstrated white feathery substances, detached cells, the absence of localized squamous epithelium, thickened intrapapillary capillary loops, and normal peristalsis ( Fig. 2 ; Video 1 ). The patient underwent complete endoscopic submucosal dissection (ESD) of both lesions ( Fig. 3 )

**Fig. 1:**
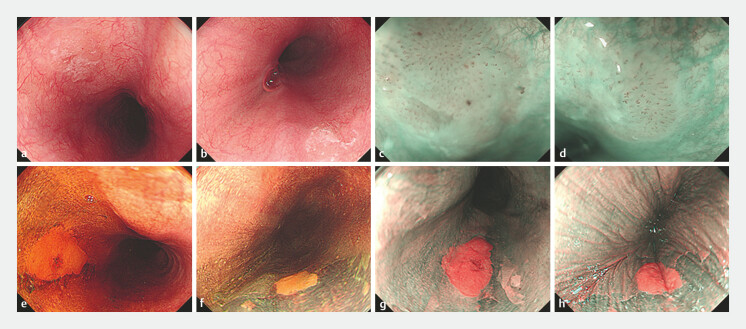
Images of an esophageal epidermoid lesion on: **a, b** white-light endoscopy; **c, d** narrow-band imaging; **e–h** Lugol chromoendoscopy.

**Fig. 2:**
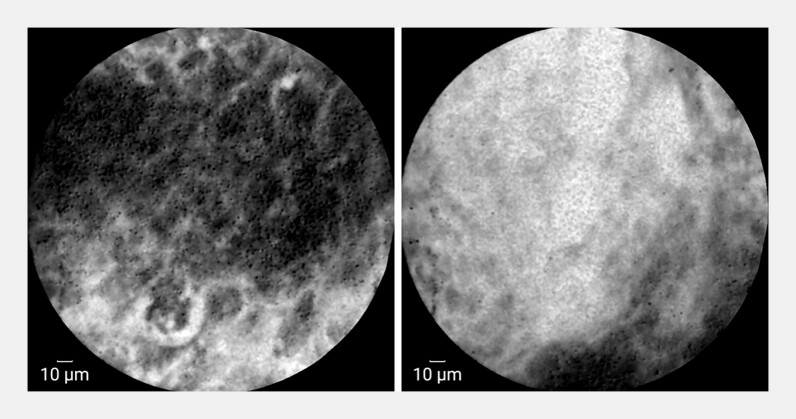
Confocal laser endomicroscopy (CLE) images showing multiple white feathery substances, as well as detached cells, local absence of squamous epithelium, and thickened intrapapillary capillary loops.

**Fig. 3:**
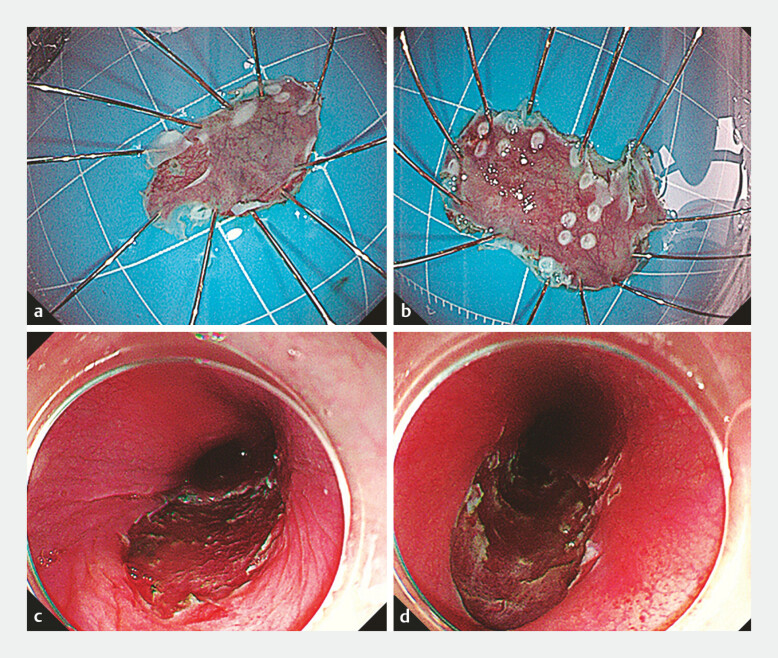
Images of the two lesions that were managed by endoscopic submucosal dissection (ESD) showing: **a, b** the macroscopic appearance of the resection specimens; **c, d** the endoscopic appearance of the post-ESD defect for: **a, c** the lesion that was 23 cm from the incisors; **b, d** the lesion that was 30 cm from the incisors.

Confocal laser endomicroscopy is performed on synchronous multiple primary early stage esophageal cancers in a 16-year-old boy.Video 1

Histopathology showed the lesion at 23 cm from the incisors to be a 0.9 × 0.6-cm squamous cell carcinoma, invading the submucosa and affecting submucous glands/ducts (200 μm), with INFb pattern ( Fig. 4 **a** ). The other lesion showed high grade dysplasia ( Fig. 4 **b** ). Immunohistochemical testing showed Ki-67 positivity in the epithelial layer. A diagnosis of synchronous multiple primary early esophageal cancer (T1aN0M0) was made. Multiple esophageal cancers have a poorer prognosis, necessitating surgery, radiotherapy, or chemotherapy 3 4 . In this case, the patient underwent radiotherapy post-ESD.

**Fig. 4:**
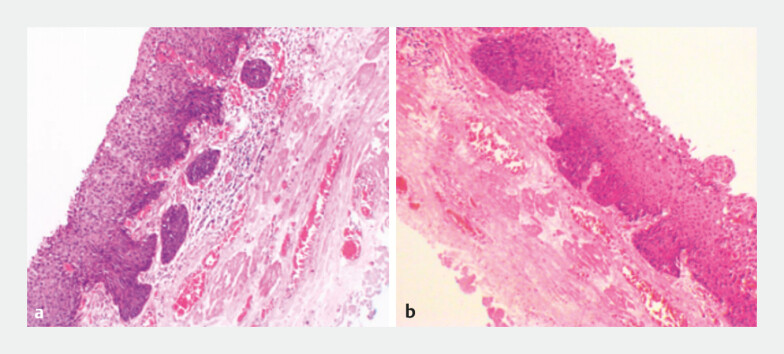
Histopathological appearance after hematoxylin and eosin (H&E) staining showing: **a** esophageal squamous cell carcinoma in the lesion that was 23 cm from the incisors; **b** high grade dysplasia in the lesion that was 30 cm from the incisors.

For patients with esophageal cancer who do not have tobacco and alcohol as risk factors, endoscopists should thoroughly examine the esophageal region for synchronous lesions. The CLE biopsy technique is safe, repeatable, and noninvasive, and demonstrates high sensitivity and specificity for early superficial esophageal squamous cell carcinoma, addressing the limitations of high definition white-light endoscopy and low resolution NBI, and the diagnostic variability of flexible indigo carmine-enhanced chromoendoscopy 5 .

Endoscopy_UCTN_Code_TTT_1AO_2AB

## Notes

Endoscopy E-Videos https://eref.thieme.de/e-videos
*E-Videos* is an open access online section of the journal *Endoscopy* , reporting on interesting cases and new techniques in gastroenterological endoscopy. All papers include a high-quality video and are published with a Creative Commons CC-BY license. Endoscopy E-Videos qualify for HINARI discounts and waivers and eligibility is automatically checked during the submission process. We grant 100% waivers to articles whose corresponding authors are based in Group A countries and 50% waivers to those who are based in Group B countries as classified by Research4Life (see: https://www.research4life.org/access/eligibility/ ). This section has its own submission website at https://mc.manuscriptcentral.com/e-videos .
